# Effects of Slaughter Knife Sharpness on Blood Biochemical and Electroencephalogram Changes in Cattle

**DOI:** 10.3390/ani10040579

**Published:** 2020-03-30

**Authors:** Jurhamid Columbres Imlan, Ubedullah Kaka, Yong-Meng Goh, Zulkifli Idrus, Elmutaz Atta Awad, Ahmed Abubakar Abubakar, Tanbir Ahmad, Hassan N. Quaza Nizamuddin, Awis Qurni Sazili

**Affiliations:** 1 Institute of Tropical Agriculture and Food Security, Universiti Putra Malaysia, Serdang 43400, Selangor, Malaysia; jurhamidimlan@yahoo.com.ph (J.C.I.); dr_ubedkaka@hotmail.com (U.K.); ymgoh@upm.edu.my (Y.-M.G.); zulidrus@upm.edu.my (Z.I.); atta.am@umk.edu.my (E.A.A.); ahmadsadeeq7@gmail.com (A.A.A.); 2 Department of Animal Science, College of Agriculture, University of Southern Mindanao, Cotabato 9407, Philippines; 3 Department of Companion Animal Medicine and Surgery, Faculty of Veterinary Medicine, Universiti Putra Malaysia, Serdang 43400, Selangor, Malaysia; 4 Department of Preclinical Sciences, Faculty of Veterinary Medicine, Universiti Putra Malaysia, Serdang 43400, Selangor, Malaysia; 5 Department of Animal Science, Faculty of Agriculture, Universiti Putra Malaysia, Serdang 43400, Selangor, Malaysia; tanbirvet05@rediffmail.com; 6 Preclinical Department, Universiti Malaysia Kelantan, Pengkalan Chepa 16100, Kelantan, Malaysia; 7 ICAR- Indian Veterinary Research Institute (IVRI), Izatnagar, Bareilly 243122, India; 8 Department of Veterinary Services, Wisma Tani, Blok Podium, Putrajaya 62630, Malaysia; quaza@dvs.gov.my; 9 Halal Products Research Institute, Universiti Putra Malaysia, Putra Infoport, Serdang 43400, Selangor, Malaysia

**Keywords:** knife sharpness, slaughter, electroencephalographic response, blood biochemical, catecholamines, animal welfare, cattle

## Abstract

**Simple Summary:**

The role of knife sharpness in slaughtering animals, from the perspective of animal welfare, is likely subconsciously ignored by the masses involved in the abattoir industry. This might be due to the difficulty in objectively quantifying the sharpness of a knife. Furthermore, a small incremental improvement in sharpness might result in a huge productivity trade-off at the abattoir when the slaughterman needs to dedicate more time to sharpen their blades in between slaughters. This study attempts to assess the effects of sharp and commercial sharp knives on the pain and stress levels of animals. After objectively measuring the sharpness of knives with an ANAGO^®^ sharpness tester, animals were slaughtered with commercial sharp and sharp knives. The results obtained from blood and brain activities related to pain and stress revealed that the two sharpness levels had different effects on the stress and pain level in animals, suggesting that the sharp knife produced the least amount of stress and pain in animals compared to those slaughtered using a commercial sharp knife.

**Abstract:**

The sharpness of the knife used for slaughter is of the utmost importance from an animal welfare perspective. The quantification of knife sharpness is almost impossible in abattoirs. The sharpness of the knife blade used to slaughter an animal, as well as its effects on animals’ pain and stress levels, is an important area of investigation that needs to be addressed. The objective of this study was to evaluate the effects of knife sharpness on blood biochemical parameters, plasma catecholamines, and electroencephalographic (EEG) responses. Twenty Brahman crossbred steers were either subjected to slaughter with a sharp knife (n = 10) or a commercial sharp knife (n = 10); knife sharpness was measured with the ANAGO^®^ sharpness tester. There was significant increase in adrenaline (*p* < 0.0001), glucose (*p* = 0.0167), creatinine kinase (*p* = 0.0123) and lactate dehydrogenase (*p* = 0.0151) at post-slaughter compared to pre-slaughter in commercial sharp knife group than in thesharp knife group. A significant increase was observed in the median frequency (*p* < 0.0001) and total power (*p* < 0.0001) of the EEG, the parameters for pain and stress, in the animals slaughtered with the commercial sharp knife than those slaughtered with the sharp knife. Thus, EEG results also supported the hormonal and biochemical results. From the results, it is concluded that animals slaughtered with a sharp knife experienced the least amount of pain and stress compared to those slaughtered with a commercial sharp knife.

## 1. Introduction

The United States Humane Slaughter Act of 1958 [1], Section 2, protects the welfare of animals by ensuring that all animals must be rendered insensible before slaughter so that that they do not feel pain. The law permits religious killings, whereby animals experience unconsciousness by cutting of a major artery, which passes up the neck and supplies blood to the head, with the use of a very sharp knife. In the Islamic method of slaughter, the animals are slaughtered by cutting the jugular vein, carotid artery, trachea, and esophagus with a sharp knife by a single swipe to incur less pain [2,3,4,5]. A similar method of slaughter has been described in Judaism, where the use of a sharp knife to cut the neck, in order to cause the least pain [6].

According to Salamano et al. (2013), Islamic dietary laws forbid the consumption of blood and dead animals but do not prohibit stunning. Furthermore, encouraging humane handling prior to and during slaughter, and stunning, which does not kill the animal, could be accepted as means for reducing suffering and meet the religious prescriptions. For instance, in Malaysia, some religious authorities accept the stunning of animals prior to neck cut [7].

One of the methods to induce quick brain death (to ensure humane slaughter) is performed by cutting the neck using a sharp knife. Animal slaughter with the use of a very sharp knife has been the usual practice. Before the advent or discovery of electric stunning instruments, the slaughter of some animal species was performed by simply striking animals with a less sharpened tool, and, at times, the draining of the blood is followed by using a less sharp knife [8]. A study conducted over the past 10 years has revealed that over 80% of people in abattoirs use inadequately sharpened knives [9]. The use of less sharp knives has been associated with reduced production as a result of injuries (musculo skeletal disorders) caused by exertion of greater force [10]. 

It is not possible to quantify the sharpness of knives at every slaughterhouse. However, an ANAGO^®^ sharpness tester can be used as an objective instrument to achieve the recommended level of knife sharpness for slaughter. The ANAGO^®^ tester provides accurate, objective, and reliable monitoring of sharpening instruments and allows sharpening to be continually and effectively controlled, optimized, and improved. The ANAGO^®^ knife sharpness tester can be used as an objective tool to measure the sharpness of a knife at commercial slaughterhouses of a very large scale [11,12,13]. There have been no studies on the effect of knife sharpness on the physiology, stress, and pain of animals. Furthermore, a dull (or less sharp) knife has been reported to require more than one cut to sever the required structures [14] for a slaughter to be considered “Halal”. More cutting of the vessels due to less sharp knives have been reported to cause false aneurysms, leading to an increase in the time needed for an animal to reach unconsciousness [15,16], leading to greater stress and pain. Studies regarding the effects of a commercial sharp knife compared to a sharp knife in similar experimental conditions have not been reported. This effect could provide insight into the effects of knife sharpness on animals’ various physiological parameters, such as blood biochemistry and stress, as well as pain. 

Following slaughter, blood parameters provide significant insight into the physiological changes associated with stress and noxious stimuli [17]. In farm animals, increased sympatho-adrenal activity stimulated by physical and psychological stress leads to hyperglycemia. This is due to the increased breakdown of glycogen in the liver [18,19]. Slaughter without prior stunning in cattle results in increased blood lactate as a result of anaerobic glycolysis. Elevated levels of creatine kinase (CK) and lactate dehydrogenase (LDH) in serum are indicative of stress, muscle damage, and muscle fatigue [20,21]. It is generally accepted that biochemical blood changes are produced in response to physical stress situations, like those induced by slaughter procedures, and these changes could compromise animal welfare [22]. The possibility of pain, stress, and the onset of unconsciousness during and after cutting of the neck are major animal welfare concerns. The pain caused by neck cutting has been a subject of debate. It has been suggested that the use of a sharp knife produces a minimal behavioral reaction and is not perceived as painful by the unstunned animal [23,24].

Pain, which is subjective by nature, has also remained a challenge to measure in animals. Since biochemical- and hormonal-based methods often have a lag time following stress-induced change, a method to quantify pain would be of great importance to animal welfare. Electroencephalography (EEG) is an established method to record the instantaneous physiological response to both stress and nociception (pain) in animals. EEG entails the recording of electrical activity via electrodes placed in various positions on the scalp [25,26,27]. Electroencephalogram spectrum changes have been used as an indicator of the experience of pain in sheep [25], red deer [28], pigs [29], cattle [30,31], dogs [32,33], and horses [34]. When animals are exposed to stressful situations, they respond through activation of their sympathetic nervous system. The activation of the first axis determines the release of adrenaline (epinephrine) and noradrenaline (norepinephrine) into the bloodstream when the animal perceives stress or painful stimuli [35]. There is a paucity of information on the effects of knife sharpness on the physiological and electroencephalographical indicators of animal welfare in cattle.

Therefore, the main objective of the present study was to determine the blood’s biochemical, hormonal, and electroencephalographic changes associated with possible noxious stimuli following slaughter via neck cutting in conscious, halal-slaughtered cattle using a sharp knife and a commercial sharp knife. The specific objectives were:

(1) To determine the physiological changes associated with neck cutting in halal slaughtered cattle using a sharp knife and a commercial sharp knife.
(2) To assess the electroencephalographic (EEG) changes associated with possible noxious stimuli following neck cutting in halal slaughtered cattle using a sharp knife and a commercial sharp knife.

## 2. Materials and Methods

### 2.1. Animals

This study was conducted following the animal ethics guidelines of the Research Policy of Universiti Putra Malaysia (UPM/IACUC/R028/2016).

**Figure 1:**
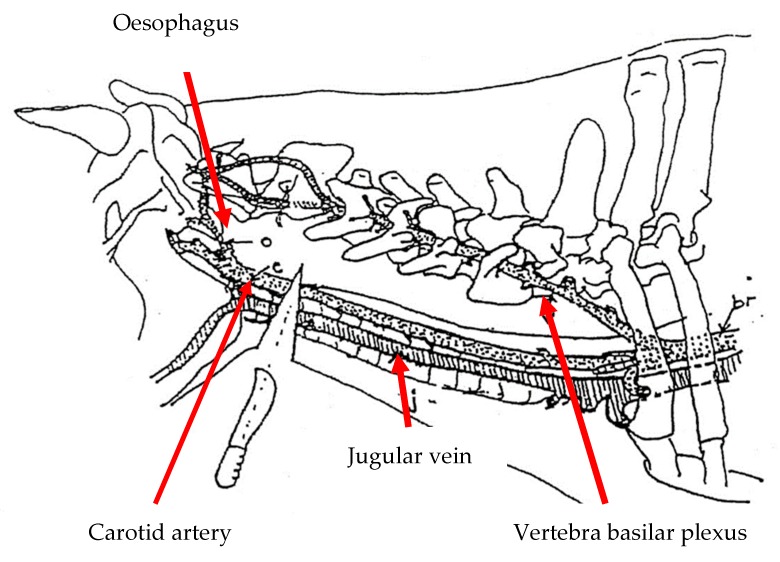
Anatomical position of the neck cut at the C1 vertebrae [35].

A total number of 20 Brahman crossbred steers, with a live weight of about 426.0 ± 24.0 kg, were obtained from Katherine, a town situated in the Northern Territory of Australia. The animals were transported by sea (for 14 d) from Darwin Port, Australia, to Pasir Gudang Port, Johor, Malaysia. Thereafter, the animals were road transported from Pasir Gudang Port to Universiti Putra Malaysia (UPM), Serdang, Selangor. The animals were fattened for five months at the Institute of Tropical Agriculture and Food Security (ITAFoS) animal facility before the road transport (30 km) to the Ruminant Commercial Abattoir, Department of Veterinary Services, Shah Alam, Selangor for slaughter. The cattle were divided into two groups comprising 10 animals each, which were either subjected to sharp knife (SK) or commercial sharp knife (CSK) cutting in a lateral recumbency slaughter position (LP). The animals were turned on their left side (90°) using a modified MARK IV restraining box following the commonly practiced Halal method of slaughter. The slaughtering of the animals was performed at the Ruminant Commercial Abattoir, Department of Veterinary Services, Shah Alam, Selangor. The slaughtering was conducted in accordance with the procedure described in MS1500: 2009 [36]. This procedure entails cutting the carotid supply routes, jugular veins, trachea, and throat. Blood parameters and electroencephalography data were acquired pre-slaughter (T1) (with the animal in the MARK IV restraint box, immediately before the box was rotated to the slaughter position) and post-slaughter (T2), after the neck cut. The cutting of the neck was carried out at the 1st cervical (C1) vertebra (Figure 1), following the requirement of OIE [14,36,37]. Before the animals were slaughtered, they were restricted from feed for 3 h with an ad libitum amount of drinking water provided.

### 2.2. Acquisition of the Slaughter Knife

**Figure 2:**
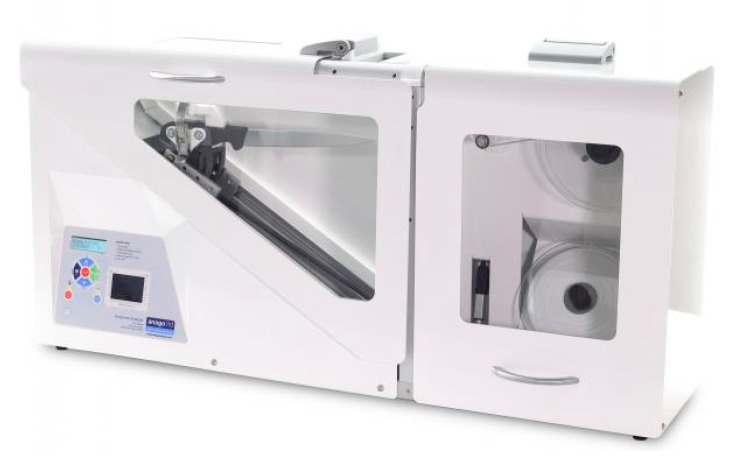
The ANAGO^®^ knife sharpness tester KST 300e.

A total of six (6) units of the Victorinox^®^ Pro cimeter knife measuring 30.48 cm were used in the experiment (Figure 2). The knives were purchased from a licensed local distributor of Victorinox^®^ Fibrox Swiss Army, Switzerland. The knife has a high carbon stainless-steel blade, which provides maximum sharpness and edge retention, a conical ground through its length and depth for a wider break point, and is ice tempered to sustain its sharpness longer. It also has patented Fibrox handles, which are textured, slip resistant, and ergonomically designed for balance and comfort and are duly approved by the National Sanitation Foundation (NSF) Ann Arbor, MI 48105, USA. A Victorinox^®^ steel rod was used to hone and maintain the sharpness of the knives throughout the duration of the experiment.

### 2.3. Measurement of Knife Sharpness

The ANAGO^®^ sharpness score is the most common way to view the results of a sharpness test. ANAGO^®^ will show the overall sharpness score out of 10 for the blade and produces a profile of sharpness from the tip to the heel of the blade. Each section of the blade is divided into blocks of 20 mm in length, from the tip of the blade to the end of the blade and categorized under sharpness zones on the vertical axis [38].

The ANAGO^®^ sharpness tester provides a blade sharpness profile. This profile gives a clear visual indication of the blade’s sharpness measured at 20-mm intervals along the length of the blade. The results are interpreted by the user to determine the sharpness of the blade and any dull/sharp areas as well as nicks in the blade. The information can assist the user in determining the corrective action required to improve the sharpness level. The results may show that the first inch of the blade from the tip end is comparatively dull compared to the rest of the blade. This would indicate that the subject is having trouble sharpening/steeling this part of the blade, and some instruction could be given to remedy the problem. The knife can then be re-tested to confirm the improvement and provide feedback on whether the changes made to the sharpening technique were effective. It is this process of experimentation that allows users to achieve significant improvements in their sharpening techniques, resulting in notable increases in average knife sharpness levels [39]. 

The control group for the current trial is the “Sharp” group with a minimum score of 8.0 based on the ANAGO Scores (Table 1). While the “Commercial Sharp” group, with an average score of 7.80, is reflective of the typical commercial slaughterman’s knife sampled in the current work. The main reason for the difference in the score between “Sharp” and “Commercial sharp” is that in order to maintain a knife above a score of 8.0 (or “Sharp”), blades have to be re-sharpened using a machine after every slaughter, whereas manual sharpening between slaughters or no sharpening at all under typical slaughter conditions could only attain an average score of 7.80, thus defined as a “Commercial Sharp” and adjudged to be acceptable for animal slaughter in the current practice by the regulating authorities. In fact, all of the knives used in the current experiment were bought new, and had a sharpness score of 7.80 upon unboxing. The researchers are also mindful that this is the lowest limit permissible based on the commercial slaughterman’s knives. Therefore, in order not to further aggravate the stress and pain of animals before and during the slaughter process, animals from the control group should have a sharpness score that is better than 7.8. The researchers are also fully aware of the significant time penalty if each of the slaughter knives has to have the sharpness of 8.0 and above. This would be a significant consideration for regulators, and industry players as the abattoir’s productivity would be affected.

### 2.4. Electroencephalography (EEG) 

Electroencephalogram activity for individual animals was recorded before the neck cut (i.e., pre-slaughter (T1) and after the neck cut (T2) using a Power Lab Biopotential Recordings system device (Power Lab data acquisition system, AD Instruments Ltd. Sydney, Australia). Upon entry to the restraining box, the animal was allowed to relax for a few seconds, the baseline blood sample was taken, and then two Kendall™ (Covidien 11c, 15 Hampshire Street, Mansfield 02048 USA) conductive adhesive hydrogel foam electrodes were placed 6–8 cm distally from the poll at an equal distance from the anterior orbital prominences of both the left and right eyes and on the left base of the poll. The EEG recordings were acquired within a band-pass signal range between 0.1 to 200 Hz, at a sampling rate of 1 kHz. These signals were then analysed offline with the help of the Chart Spectral Analysis function of Chart 5.0™ (Powerlab™ data acquisition system Sydney, Australia). Prior to EEG analysis, the raw EEG recordings were resampled at 1024 Hz, and only frequencies between 0.1 to 30 Hz were obtained to minimize the presence of artefacts. Possible interferences from concurrent electrocardiography signals were digitally removed from the raw EEG recordings using the Chart 5.0 software (AD Instruments) before analysis. The signals were then processed in blocks of 1 s epochs, yielding 60 epochs per minute. The signal was then filtered into band-pass filters to yield delta (0.1–4 Hz), theta (4.1–8 Hz), alpha (8.1–12 Hz), and beta (12.1–20 Hz) waves. The Chart Spectral Analysis Function (Chart 5.0 software, AD Instruments) was used to analyse each frequency component. Briefly, the signals were subjected to fast Fourier transformation (FFT) and power–density curves for each frequency band were derived on the basis of cosine bell distribution. Each calculation of the alpha, beta, delta, and theta waves was done for the pre-slaughter and post-slaughter root mean square (RMS). The median frequency (F50; the frequency below which 50% of the total power of the EEG) and total power (Ptot; the total area under the power spectrum curve) at pre-slaughter and post-slaughter were also determined.

### 2.5. Blood Sampling

Ten milliliter blood samples were collected with the use of 18-gauge needles via jugular venipuncture with an aseptic precautionary measure. The blood samples were collected at pre-slaughter in the restraint box (T1), followed by a post-neck cut (from blood flow) (T2). The blood samples for biochemical analyses were collected into a vacutainer (BD Franklin Lakes, NJ, USA), stored in the ice, and taken to the Clinical Pathology and Hematology Laboratory, Faculty of Veterinary Medicine, Universiti Putra Malaysia after about an hour. The blood samples required for catecholamines (adrenaline and noradrenaline) or hormonal analyses were collected into vacutainer K3 ethylene diamine tetra acetic acid (EDTA) tubes and slanted in crushed ice, after which the samples were centrifuged at 800 rpm, 4 °C for 15 min. The retrieved plasma portion was separated into 2 mL aliquots and kept at −80 °C until the analysis.

### 2.6. Determination of Blood Biochemical Parameters

Lactate, glucose, urea, total protein, creatinine, creatine kinase (CK), calcium, and lactate dehydrogenase (LDH) were determined with the use of a programmed analyzer (Auto Analyzer Hitachi 902, Tokyo, Japan). All reagents used in the analysis were obtained from Roche (Hitachi 902, Tokyo, Japan).

### 2.7. Determination of Adrenaline

The adrenaline (epinephrine) content in the blood was analyzed quantitatively with the use of an Adrenaline Plasma Enzyme-Linked Immuno Sorbent Assay (ELISA) High Sensitive kit # BA E-4100 (LDN^®^, Germany). The competitive ELISA kit uses the microtiter plate format. The adrenaline was extracted from a plasma sample with the help of a cis-diol-specific affinity gel, acylated, and then modified enzymatically. The antigen was bound to the solid phase of the microtiter plate, and the derivatized standards, controls, samples, as well as the solid phase bound analytes, competed for a fixed number of anti-serum binding sites.

#### 2.7.1. Sample Preparation, Extraction, and Acylation

Twenty-five microliters of standards and controls, as well as 400 µL of plasma samples, were pipetted into the respective wells of the extraction plate. A total of 500 µL of deionized water was added to the wells with standards and controls, while 200 µL of deionized water was added to the wells with samples. The contents were mixed by shaking the plate on a microplate shaker for 1 min. Thereafter, 25 µL of Tris EDTA buffer was pipetted into all wells, and the plate was covered with adhesive foil and incubated at room temperature (20–25 °C) for 60 min on a plate shaker (MS Major Science, Taiwan) at 600 rpm. Following incubation, the foil was removed, and the plate was blot dried by inverting it and tapping it on a clean lint-free towel. One millilitre of wash buffer was pipetted into all wells, and the plate was shaken at 600 rpm on a plate shaker for 5 min at room temperature. The plate was then blot dried by turning it over and tapping on a neat tissue towel. The washing was repeated for one time. After washing, 150 µL of acylation buffer followed by 25 µL of acylation reagent were pipetted into all wells. In order to ensure rapid addition, an eight-channel pipette (Biopette ATM, Poland) was used. The incubation of the plate for 20 min was carried out at room temperature, which was done on a plate shaker at 600 rpm. The content in the plate was then evacuated completely and blot dried by turning over and tapping on a clean lint-free towel. One millilitre of wash buffer was pipetted into all wells, and the plate was shaken for 5 min at room temperature. Blot drying of the plate was done by turning the plate over and tapping it on a neat tissue towel. The washing was then repeated. Following the washing, 100 µL of HCl was pipetted into all wells using an 8-channel pipette. A foil was used to cover the plate, and the plate was incubated on a plate shaker at 600 rpm for 10 min at room temperature.

#### 2.7.2. Enzymatic Conversion

Using the above contents of the extraction plate, 90 µL of the extracted standards, control, and samples were pipetted into the various wells of the microtiter plate. Using an 8-channel pipette, 25 µL of the enzyme solution was added to all wells. The plate was then shaken for 1 min on a shaking incubator after covering it with foil. Lastly, the plate was incubated (Shaker Incubator, Hotech^®^ 702R, Taipei, Taiwan) for 2 h at 37 °C. 

### 2.8. Adrenaline Evaluation

Using the contents of the enzyme plate above (100 µL of standards), the control and samples were pipetted into their respective pre-coated Adrenaline Microtiter Strips (LDN^®^, Nordhorn, Germany). Using an 8-channel pipette, 50 µL of the respective Adrenaline antiserum was added to all wells. At room temperature, the plate was incubated for 1 min on a shaker at 600 rpm after covering it with foil. This was followed by the incubation of the plate overnight (15–20 h) at 4 °C. The next day, the foil was removed, and the contents of the plate were discarded. Each well was thoroughly washed with 300 µL wash buffer 4 times and then blot dried by tapping and turning over the plate on clean tissue towels. Thereafter, 100 µL of the substrate was then included in all wells. Incubation of the plate was then done at room temperature for 25 min on a plate shaker at 600 rpm while preventing it from being exposed to sunrays. Finally, 100 µL of stop solution was pipetted into all wells, and the absorbance was read in less than 10 min with the use of an auto UV Xenon flash lamp micro plate reader (infinite M200, Tecan, Austria) set to 450 nm, with a reference wavelength between 620 and 650 nm. Absorbance readings of the standards (linear, y-axis) were plotted against the corresponding standard concentrations (logarithmic, x-axis) to obtain the calibration curve. A non-linear regression (4 parameters) was adopted for curve fitting. The plasma sample concentration was determined from the standard curve, which was then multiplied by the volume factor to account for dilution during the extraction. The volume factor was derived from the following equation:

Volume factor = (600 µL)/(used plasma volume (µL)).

### 2.9. Determination of Noradrenaline

The quantitative analysis of noradrenaline (norepinephrine) content in the blood was done using a Noradrenaline Plasma ELISA High Sensitive kit # BA E-4200 (LDN^®^, Nordhorn, Germany). This kit works under the same principle as that explained above for adrenaline. The sample preparation, extraction, and acylation, as well as enzymatic conversion, were carried out following a procedure similar to the one discussed above.

Ten microliters of the standards, control, and samples were pipetted from the plate of the enzyme into the individual pre-coated Noradrenaline Microtiter Strips. Using an 8-channel pipette, 50 µL of the Noradrenaline antiserum was added to all wells. The steps that followed (i.e., covering with foil through calculation of noradrenaline concentration) were similar to those described in the section for adrenaline.

### 2.10. Data Analysis

The analysis was carried out with a factorial design. Statistical analysis was performed using the General Linear Model (GLM) procedure of the Statistical Analysis System (SAS) package Version 9.4 (SAS Institute Inc., Cary, NC, USA). Analysis of data was done using the sampling time and slaughter knife as the main effects within the ANOVA procedure. When noticeable effects were seen, a comparison of means was done using Duncan’s multiple range test. The statistical significance was set at *p* < 0.05. 

## 3. Results

### 3.1. Blood Biochemical Parameters 

The animals slaughtered with a sharp knife had higher glucose levels than those slaughtered with a commercial sharp knife. On the other hand, creatine kinase, lactate dehydrogenase, and lactate were higher in animals slaughtered with a commercial sharp knife. Blood biochemistry alterations, as affected by the slaughter technique, are shown in Table 2.

In this study, non-significant (*p* > 0.05) interactions were noticed between knife sharpness and the sampling point for glucose, creatine kinase, lactate dehydrogenase, calcium, total protein, creatinine, and lactate. Glucose concentration was not different (*p* > 0.05) between pre and post-slaughter within the sharp knife group. However, a significant difference (*p* = 0.0167) was observed between pre and post-slaughter for animals slaughtered with a commercial sharp knife. The glucose concentration was higher in animals subjected to slaughter with a sharp knife. At pre-slaughter, the sharp knife group resulted in a greater (*p* = 0.0001) glucose level (5.21 ± 0.10) compared to the samples obtained from the animals subjected to a commercial sharp knife (4.44 ± 0.05). 

Creatine kinase did not differ between the pre and post-slaughter times in the sharp group animals. The creatine kinase values were greater (*p* = 0.0057) in animals slaughtered with a commercial sharp knife than in animal slaughtered with a sharp knife post-slaughter (753.30 ± 21.39 vs. 449.60 ± 94.49). Lactate dehydrogenase significantly increased (*p* < 0.05) post-slaughter compared to pre-slaughter (1137.90 ± 47.86 vs. 1021.40 ± 18.68) in animals slaughtered with a sharp knife and (2122.60 ± 95.03 vs. 1639.70 ± 152.55) in animals slaughtered with a commercial sharp knife. Lactate dehydrogenase values were greater in animals slaughtered with a commercial sharp knife compared to those slaughtered with a sharp knife pre-slaughter (1639.70 ± 152.55 vs. 1021.40 ± 18.68) and post-slaughter (2122.60 ± 95.03 vs. 1137.90 ± 47.86). 

Neither sharpness nor slaughter times had any effect on the calcium levels in both groups of animals slaughtered with sharp or commercial sharp knives. A similar trend was observed for total protein and creatinine. A significant difference (*p* = 0.0288) in lactate was noted between the two knife sharpness levels at pre-slaughter (8.82 ± 1.41 vs. 4.94 ± 0.80). 

A significantly lower level (*p* = 0.0167) of glucose was indicated by the samples obtained at pre-slaughter (4.44 ± 0.05) than the level obtained at post-slaughter (4.83 ± 0.13), and this was only seen in the animals assigned to the commercial sharp knife group. Although not significant, in the case of a sharp knife, the glucose level tended (*p* = 0.9193) to be lower in the pre-slaughter (5.21 ± 0.10) samples than in the post-slaughter ones (5.23 ± 0.16). No significant difference (*p* = 0.4951) in lactate was indicated by the samples obtained from the commercial sharp knife group at post-slaughter (10.17 ± 1.32) compared to those obtained at pre-slaughter (8.82 ± 1.41). A significantly greater level (*p* = 0.0102) of lactate was observed in the samples obtained from the sharp knife group at post-slaughter (8.05 ± 0.72) than those obtained at pre-slaughter (4.94 ± 0.80). A greater LDH level (*p* = 0.0151) was recorded in the post-slaughter blood samples (2122.60 ± 95.03) than in the pre-slaughter (1639.70 ± 152.55) blood samples obtained from the commercial sharp knife group. Likewise, a higher LDH level (*p* = 0.0359) was also recorded in the post-slaughter blood samples (1137.90 ± 47.86) than the level in the pre-slaughter (1021.40 ± 18.68) blood samples obtained from the sharp knife group.

### 3.2. Influence of Knife Sharpness on Hormonal Parameters

In this study, a significant interaction was observed between the treatment and sampling points for adrenaline (*p* < 0.0001). The concentration of adrenaline was affected by the knife sharpness, which was evidenced by the greater concentrations of adrenaline (*p* < 0.0001) in animals assigned to the commercial sharp knife group than those of the sharp knife group at post-slaughter (1222.09 ± 14.77 vs. 1053.96 ± 17.97). The concentrations of adrenaline differed significantly between the pre-slaughter and post-slaughter sampling time, and these concentrations were found in both groups of knife sharpness. Greater concentrations of adrenaline (*p* < 0.0001) were recorded in blood samples obtained at post-slaughter than those collected at pre-slaughter for the animals in the commercial sharp knife (1222.09 ± 14.77 vs. 732.78 ± 2.69) and sharp knife (1053.96 ± 17.97 vs. 728.01 ± 1.51) groups. No significant interactions (*p* = 0.2974) between knife sharpness and sampling period were noted for plasma noradrenaline concentration. At post-slaughter, the concentrations of noradrenaline were significantly greater in animals slaughtered with a commercial sharp knife than those slaughtered with a sharp knife (482.37 ± 11.24 vs. 438.17 ± 6.77; *p* = 0.0072) (Table 3).

### 3.3. Influence of Knife Sharpness on EEG Recording

Significant interactions (*p* < 0.05) between the knife sharpness and sampling time point were observed for alpha, beta, theta, and F50, while no significant interaction (*p* > 0.05) between the knife sharpness and sampling period was seen for the delta or Ptot. Before slaughter, no significant difference was recorded between the alpha, delta, theta, Ptot, and F50 for animals slaughtered with sharp and commercial sharp knives, except for the beta wave. Conversely, after slaughter, greater values among the animals slaughtered with a commercial sharp knife compared to those slaughtered with a sharp knife were recorded for the alpha group (7.45 ± 0.32 vs. 6.02 ± 0.26), delta group (56.91 ± 1.67 vs. 48.41 ± 1.67), theta (12.67 ± 0.66 vs. 9.15 ± 0.44), Ptot (79.09 ± 1.05 vs. 72.59 ± 1.33), and F50 (30.94 ± 1.39 vs. 20.25 ± 1.47). In animals slaughtered with a sharp knife, significantly greater values (*p* < 0.05) were observed at post-slaughter compared to pre-slaughter for alpha (6.02 ± 0.26 vs. 2.48 ± 0.19), beta (10.38 ± 0.34 vs. 4.63 ± 0.30) delta (48.41 ± 1.69 vs. 19.03 ± 1.63), theta (9.15 ± 0.44 vs. 3.25 ± 0.32), Ptot (72.59 ± 1.33 vs. 27.38 ± 1.92), and F50 (20.25 ± 1.47 vs. 16.74 ± 0.88). Similarly, animals slaughtered with a commercial sharp knife had significantly greater values (*p* < 0.05) at post-slaughter compared to pre-slaughter for alpha (7.45 ± 0.32 vs. 2.76 ± 0.12), beta (10.95 ± 0.36 vs. 6.86 ± 0.32), delta (56.91 ± 1.67 vs. 16.57 ± 0.88), theta (12.67 ± 0.66 vs. 3.22 ± 0.15), Ptot (79.09 ± 1.05 vs. 28.48 ± 1.10), and F50 (30.94 ± 1.39 vs. 18.41 ± 0.78) (Table 4).

## 4. Discussion

### 4.1. Blood Biochemical Parameters

There was a significant difference between the pre- and post-slaughter glucose, CK, and LDH levels in animals slaughtered with a commercial sharp knife compared to those slaughtered with a sharp knife. Likewise, the results of the study showed that the use of a commercial sharp knife significantly increased the levels of catecholamine (adrenaline and noradrenaline) compared to the use of a sharp knife. Similarly, the EEG parameters associated with pain increased significantly in animals slaughtered with a commercial sharp knife compared to those slaughtered using a sharp knife. The results of this research indicate that blood glucose, CK, and LDH levels increased post-slaughter. This points to an association between plasma glucose increase and the stimuli associated with the slaughter process, which could be due to the fact that the cut made during slaughter is a painful stimulus that stimulates the structures of the nervous system that respond to the sensitivity of pain and emotion. This stimulation also activates the pituitary-adrenal axis and sympathetic nervous system. The influence of this process on the body includes an acceleration of the heart rate and respiration rate, a rise in body temperature, and the recirculation of visceral blood volume to the skeletal muscle and brain. Moreover, the quality of the cut affects bleeding, the rate of unconsciousness, and signs of life [39].

These results further support the finding that when animals are exposed to stressful conditions, they secrete catecholamine and glucocorticoids, which enhance hepatic glycogenolysis, thereby leading to high glucose levels [40,41]. The study in [3,4] showed that temporal stress increases glucose and lactate levels regardless of the abrupt increase of both values following a stressful handling event [42,43]. Moreover, increased levels of creatine kinase and lactate dehydrogenase were observed in this study. Creatine kinase is an enzyme whose sensitivity is increased during extraneous activity, due to its effect on catalyzing the transformation of creatine to phosphocreatine for the energy reservoir in tissues. The increased levels of CK in the blood are an indication of how stressful the handling facilities were before the animal was slaughtered, as well as the extent of muscular damage during handling [44,45,46]. This enzyme occurs in different tissues, and its occurrence in blood plasma indicates the degree of muscular damage [47]. Lactate dehydrogenase is an enzyme found in the liver whose activity is altered in response to factors related to stress or a worn muscle. Creatine kinase and LDH are enzymes that are released into the blood when there is muscle damage, thereby indicating stress and fatigue [48]. Cattle react quickly to stressful settings, resulting in an increased concentration of hormones (i.e., adrenaline and noradrenaline), as well as enzymes (i.e., creatine kinase and lactate dehydrogenase) [49]. The presence of loud noises in the environment where slaughtering is carried out is also another essential stress factor. The amount of blood lactate, as well as the creatine kinase activity in cattle, was significantly affected by the intensity of the squealing sound [50,51]. Previous studies also found that changes in the plasma activities of creatine kinase and lactate dehydrogenase activities can be used as indicators of pre-slaughter stress [52,53].

The results of the present study and the line of evidence from the previous studies demonstrate that levels of creatine kinase, lactate dehydrogenase, and lactate are increased in animals slaughtered using a commercial sharp knife than those in animals slaughtered using a sharp knife. This suggests that animals slaughtered with a commercial sharp knife experience higher levels of stress than those slaughtered with a sharp knife.

### 4.2. Influence of Knife Sharpness on Hormonal Parameters

It is well established that animals that are under stressful conditions respond by activating the sympathetic and hypothalamic–pituitary–adrenal axes. The activation of the first axis influences the release of epinephrine and norepinephrine into the blood stream as a preparatory step when an animal observes a problem and prepares its immediate response [35].

The results of this study show that using a commercial sharp knife significantly increased catecholamine levels (adrenaline and noradrenaline). It is, therefore, assumed that a cut from a commercial sharp knife is painful for animals, as nociceptors in the region of the incision are triggered. This cut is further followed by exsanguination, resulting in a drop in blood pressure, which triggers the sympathetic adrenal medullary nervous system, leading to the release of epinephrine and norepinephrine from the sympathetic endings [54]. Several factors that can enhance pain perception include making several cuts, changes in the direction of the cut, the use of a blunt blade, and a positioning of the neck that negatively affects a good cut or impedes the flow of the blood [55,56]. Nonetheless, under stress, the high concentration of catecholamines that is released into the bloodstream prepares the animal for rapid energy loss [40].

A previous study on plasma catecholamines in severely injured patients indicated that multiple injuries in a person initially produced a pronounced increase in both adrenaline and noradrenaline levels, which is attributed to the increased excretion by the adrenal medulla caused by hypovolemia and partly to the release of noradrenaline from the sympathetic nerve endings caused by tissue hypoxia and acidosis [57]. The total plasma catecholamine measured in patients after the injury were almost doubled on the eighth day and remained significantly higher for 6 h after the trauma [57]. The stimulating effect of pain and various other stress factors on the sympathetic nervous system resulted in persistently high plasma noradrenaline levels in critically injured patients. Pain is considered a powerful provoker of the sympathoadrenal axis, which increases sympathetic tone and catecholamine release [58]. In this study, significantly higher noradrenaline levels were observed in cattle slaughtered using a commercial sharp knife, which is believed to induce more stress and pain in the animals compared to those slaughtered with a sharp knife. 

### 4.3. Influence of Knife Sharpness on EEG Recording

In this study, the EEG parameters increased significantly after slaughter compared to pre-slaughter in both groups of animals slaughtered with sharp and commercial sharp knives. However, this increment was significantly higher in the commercial sharp group than in the sharp group. Sabow et al. (2017) reported that alpha, beta, delta, and theta increased significantly post-slaughter compared to pre-slaughter in goats [59]. In another study on cattle, reported by Zulkifli, et al. (2014), alpha and beta increased significantly after the neck cut [31]. Not much literature is available regarding the EEG frequencies caused by neck cuts on animals that have not been stunned. Therefore, more research is warranted to look at the status of these waves in animals in response to various stressors. 

The median frequency and Ptot are reported as indicators of pain and stress associated with pain in animals [4,30,31,59]. The literature regarding the MF and Ptot of EEGs has been reported in various studies [4,25,28,31,32,33,59]. In this study, F50 and Ptot increased significantly in response to neck cuts compared to their pre-slaughter values. Similar results have been reported in response to slaughter in goats [59], cattle [31], and calves [4]. An increase in F50 and Ptot in response to noxious stimulation has been reported in dogs [32,33,60], sheep [25,61], ponies [62], and red deer [63]. It has been suggested that the use of a very sharp knife is not perceived as painful by the animals [64]. In this study, the rise in the F50 and Ptot after the neck cut was significantly higher in the commercial sharp group than in the sharp group, suggesting that the sharp knife is less painful to the animals than the commercial sharp knife. 

Electroencephalogram is reported to be the most reasonable method for evaluating unconsciousness, as it shows the underlying electrical activity of populations of neurons supported by glia cells [27]. EEGs indicate characteristic changes when animals are unconscious. Analysis of these electroencephalographic changes gives insight into the degree of nociception and stress that animals experience at the point of slaughter [4]. 

Electroencephalography is an established neurophysiological technique to evaluate pain and nociception in animals. Alpha and beta waves are high frequency fast waves, whereas delta and theta are low frequency slow waves. Detecting the perception of noxious stimuli in the brain typically begins with arousal or desynchronization. Desynchronization is a typical EEG response characteristic of nociception [31,32]. In humans, alpha waves have been thought to indicate relaxed awareness without any attention or concentration. Beta waves are the usual waking rhythm of the brain associated with active thinking, active attention, focus on the outside world, or solving concrete problems and are found in normal adults. Higher beta waves may be recorded when a human is in a state of panic [65,66]. Delta waves are related to deep sleep. Theta waves have been related with access to unconscious material, creative inspiration, and deep meditation [67]. 

In this study alpha, beta, delta, and theta waves increased significantly in cattle after slaughter. The association of pain with an increase in alpha, beta, and delta waves has been reported in humans, and a decrease in these waves was associated with a reduction in pain by carbamazepine treatment [68]. Similarly, a perception of pain by the immersion of the hands in cold water increased delta and beta waves [69]. For dogs, alpha and beta bands have been reported to increase, while delta decreased, in response to surgery [70]. Although there is little discrepancy in various waves between different studies, this lack of discrepancy might be due to the different experimental conditions and needs to be further investigated. Nevertheless, these results are in line with results of our study in general. Thus, these results support the results in our study that slaughter affects various EEG waves. Furthermore, the significantly higher values in the commercial sharp group compared to the sharp group suggests that the commercial sharp knife was more painful than the sharp knife.

Slaughtering animals through a ventral neck incision followed by exsanguination is seen by several religious faiths, such as Islam and Judaism, as the proper method of slaughtering animals to be consumed [22]. The pain resulting from this neck cut has been the topic of many discussions. It has been suggested that using an exquisitely sharp knife results in minimal behavioral reactions in animals, and, due to this, a neck cut is often perceived as being non-painful to the animal [23,71]. Accordingly, in the present study, we found that the biochemical, hormonal, and EEG parameters related to stress and pain were higher in animals slaughtered with a commercial sharp knife than in those slaughtered with a sharp knife. Furthermore, the hormonal parameters of stress supported the EEG parameters of stress and pain. This suggests that animals slaughtered with a sharp knife experienced less stress and pain than those slaughtered with a commercial sharp knife.

A wide range of attitudes to animals by people involved in handling during transport (handlers, managers) have a negative effect on animals. Loading animals for haulage is an inevitable process in the production chain, which is a requisite for animals to go through at one stage of their lives. Grandin (1997) reported that fear and novelty are major stressors in ruminants and that these animals respond to changes in their environment and view people and facilities around as predators. However, animals do adapt to non-invasive treatments, such as restraint in a chute for blood collection [43,72]. According to Grandin (1997) and Ferguson and Warner (2008), as a result of pre-slaughter challenges, animals may experience fear due to novelty. Bruising due to physical injury during pre-slaughter handling has been identified as it occurs prevalently [72,73]. In the current study, animals were transported using the highway at night to minimize the effect of thermal extremes and skilled personnel were involved in handling, restraint, and slaughter in order to reduce the risk of introducing man-made challenges that can largely be avoided.

## 5. Conclusions

The current results show that while the slaughter process is stressful to the animal, the sharpness of the blades used during slaughter could influence the intensity of the animals’ stress responses. The catecholamines, glucose, and liver enzymes were higher after the neck cut in animals slaughtered with a commercial sharp knife. Likewise, the EEG profiles indicated that animals may have endured less pain when slaughtered using a sharp knife. The sharpness of the knife is an important factor to minimize pain during slaughter. Therefore, it is important that knives are strictly checked for sharpness before slaughter.

## Figures and Tables

**Table 1:** Change in force required to use a knife/blade relative to a knife that scores 8.0 on the ANAGO^®^ sharpness scale [37].

ANAGO Score		Relative Force Required to Cut
10.0	=	no force required
9.7	=	a tenth of the force required
9.5	=	less than a fifth of the force
9.0	=	less than half the force
8.5	=	two-thirds of the force
8.0	=	1 x force
7.5	=	a third more force
7.0	=	four-fifths more force, nearly twice as much
6.5	=	two and a half times as much force
6.0	=	more than three times as much force
5.5	=	four times as much force
5.0	=	nearly five and a half times as much force
4.5	=	seven times as much force
4.0	=	more than nine times as much force
3.5	=	13 times as much force
3.0	=	18 times as much force
2.0	=	42 times as much force

**Table 2:** Differences in the blood’s biochemical parameters in cattle subjected to different knife sharpness.

Parameter	Treatment	Sampling Period			
		Pre-slaughter	Post-slaughter	*p*-value	Trt * Period
Glucose	Sharp	5.21 ± 0.10 ^a,x^	5.23 ± 0.16 ^a,x^	0.9193	0.1387
(mmol/l)	Commercial sharp	4.44 ± 0.05 ^b,y^	4.83 ± 0.13 ^a,x^	0.0167	
	*p*-value	<0.0001	0.0747		
Creatine kinase	Sharp	448.20 ± 87.73 ^a,x^	449.60 ± 94.49 ^a,y^	0.9915	0.1636
(U/l)	Commercial sharp	538.10 ± 74.31 ^b,x^	753.30 ± 21.39 ^a,x^	0.0123	
	*p*-value	0.4445	0.0057		
Lactate	Sharp	1021.40 ± 18.68 ^b,y^	1137.90 ± 47.86 ^a,y^	0.0359	0.0578
Dehydrogenase	Commercial sharp	1639.70 ± 152.55 ^b,x^	2122.60 ± 95.03 ^a,x^	0.0151	
(U/l)	*p*-value	.0008	<0.0001		
Calcium	Sharp	2.06 ± 0.13 ^a,x^	2.04 ± 0.06 ^a,x^	0.8955	0.6986
(mmol/l)	Commercial sharp	2.12 ± 0.10 ^a,x^	2.03 ± 0.11 ^a,x^	0.4827	
	*p*-value	0.7311	0.8597		
Total protein	Sharp	75.49 ± 6.54 ^a,x^	78.09 ± 3.15 ^a,x^	0.7244	0.5355
(g/l)	Commercial sharp	82.90 ± 5.32^ax^	79.20 ± 4.50 ^a,x^	0.6023	
	*p*-value	0.3911	0.8424		
Creatinine	Sharp	167.40 ± 9.67 ^a,x^	294.90 ± 128.54 ^a,x^	0.3357	0.4685
(µmol/l)	Commercial sharp	171.20 ± 12.21 ^a,x^	203.50 ± 10.82 ^a,x^	0.0633	
	*p*-value	0.8101	0.4877		
Lactate	Sharp	4.94 ± 0.80 ^b,y^	8.05 ± 0.72 ^a,x^	0.0102	0.4332
(mmol/l)	Commercial sharp	8.82 ± 1.41 ^a,x^	10.17 ± 1.32 ^a,x^	0.4951	
	*p*-value	0.0288	0.1761		

^a,b^ Means within the same row with different superscripts are significantly different at *p* < 0.05; ^x,y^ Means within the same column with different superscripts are significantly different at *p* < 0.05.

**Table 3:** Changes in catecholamine parameters in cattle subjected to different knife sharpness.

Parameter	Treatment	Sampling Period			
		Pre-slaughter	Post-slaughter	*p*-value	Trt * Period
Adrenaline	Sharp	728.01 ± 1.51 ^b,x^	1053.96 ± 17.97 ^a,y^	<0.0001	<0.0001
(pg/mL)	Commercial sharp	732.78 ± 2.69 ^b,x^	1222.09 ± 14.77 ^a,x^	<0.0001	
	*p*-value	0.1535	<0.0001		
Noradrenaline	Sharp	435.07 ± 3.12 ^a,x^	438.17 ± 6.77 ^a,y^	0.6871	0.2974
(pg/mL)	Commercial sharp	459.54 ± 12.5 ^a,x^	482.37 ± 11.24 ^a,x^	0.2057	
	*p*-value	0.0881	0.0072		

^a,b^ Means within the same row with different superscripts are significantly different at *p* < 0.05; ^x,y^ Means within the same column with different superscripts are significantly different at *p* < 0.05.

**Table 4:** Electroencephalographic changes in cattle subjected to different knife sharpness at pre- and post-slaughter.

Parameter	Treatment	Sampling Period			
		Pre-slaughter	Post-slaughter	*p*-value	trt*period
Alpha (µv)	Sharp	2.48 ± 0.19 ^b,x^	6.02 ± 0.26 ^a,y^	<0.0001	0.0026
	Commercial sharp	2.76 ± 0.12 ^b,x^	7.45 ± 0.32 ^a,x^	<0.0001	
	*p*-value	0.2200	0.0006		
Beta (µv)	Sharp	4.63 ± 0.30 ^b,x^	10.38 ± 0.34 ^a,x^	<0.0001	0.0003
	Commercial sharp	6.86 ± 0.32^by^	10.95 ± 0.36 ^a,x^	<0.0001	
	*p*-value	<0.0001	0.2522		
Delta (µv)	Sharp	19.03 ± 1.63 ^b,x^	48.41 ± 1.69 ^a,y^	<0.0001	0.1323
	Commercial sharp	16.57 ± 0.88 ^b,x^	56.91 ± 1.67 ^a,x^	<0.0001	
	*p*-value	0.1842	0.0004		
Theta (µv)	Sharp	3.25 ± 0.32 ^b,x^	9.15 ± 0.44 ^a,y^	<0.0001	0.0009
	Commercial sharp	3.22 ± 0.15 ^b,x^	12.67 ± 0.66 ^a,x^	<0.0001	
	*p*-value	0.9342	<0.0001		
Ptot (µv)	Sharp	27.38 ± 1.92 ^b,x^	72.59 ± 1.33 ^a,y^	<0.0001	0.0948
	Commercial sharp	28.48 ± 1.10 ^b,x^	79.09 ± 1.05 ^a,x^	<0.0001	
	*p*-value	0.6217	0.0002		
MF (µv)	Sharp	16.74 ± 0.88 ^b,x^	20.25 ± 1.47 ^a,y^	0.042	<0.0001
	Commercial sharp	18.41 ± 0.78 ^b,x^	30.94 ± 1.39 ^a,x^	<0.0001	
	*p*-value	0.156	<0.0001		

^a,b^ Means within the same row with different superscripts are significantly different at *p* < 0.05; ^x,y^ Means within the same column with different superscripts are significantly different at *p* < 0.05.
